# Evaluation of problematic screen exposure in pre-schoolers using a unique tool called “seven-in-seven screen exposure questionnaire”: cross-sectional study

**DOI:** 10.1186/s12887-021-02939-y

**Published:** 2021-10-25

**Authors:** S. Songül Yalçin, Özlem Tezol, Nilgün Çaylan, Meryem Erat Nergiz, Deniz Yildiz, Şeyma Çiçek, Ayşe Oflu

**Affiliations:** 1 Department of Pediatrics, Hacettepe University, Faculty of Medicine, Ankara, Turkey; 2 Department of Pediatrics, Mersin University, Faculty of Medicine, Mersin, Turkey; 3 Department of Child and Adolescents Health, Ministry of Health, Ankara, Turkey; 4 Department of Pediatrics, Yıldırım Beyazıt University, Yenimahalle Research Hospital, Ankara, Turkey; 5 Department of Pediatrics, Dr. Sami Ulus Child Hospital, Ankara, Turkey; 6 Department of Pediatrics, Etimesgut Şehit Sait Ertürk Hospital, Ankara, Turkey; 7 Department of Pediatrics, Afyon Health Sciences University, Faculty of Medicine, Afyon, Turkey

**Keywords:** Problematic screen exposure, Pre-schoolers, Unique, Questionnaire

## Abstract

**Background:**

Screen media exposure has been increasing in the preschool years. Risky aspects of screen exposure have many potential negative effects on children’s health. We aimed to evaluate problematic screen exposure in Turkish preschool children by using a unique tool called the “Seven-in-Seven Screen Exposure Questionnaire” and to investigate factors associated with problematic screen exposure.

**Methods:**

A questionnaire form was designed including general descriptive questions in the first part. In the second part, a questionnaire we designed called the “Seven-in-Seven Screen Exposure Questionnaire” was conducted to evaluate problematic screen exposure characteristics. The questionnaire included seven items: daily screen time, viewing with parent(s), setting screen limits, screen exposure during meals and in the hour before bedtime, age of onset of screen exposure, and viewing low-quality content. The total problematic screen exposure score (range 0–13) was generated by summing scores from the seven items. Total scores are classified into two categories: low (< 7) and high (≥ 7). Logistic regression was performed to search for independent parameters associated with problematic screen exposure.

**Results:**

One thousand two hundred forty-five mother-child pairs participated in this study. The median age of the children was 3.9 (IQR: 2.9–4.7) years and 51% were males. Overall, 280 children (22.5%) had a problematic screen exposure score of ≥7 (high). The median problematic screen exposure score was 4 (IQR: 3–6). Maternal age of < 30 years; paternal age of ≥30 years; maternal educational level of ≤12 years; the age of 24–48 months; home-based daycare; postponing eating, toileting, or sleeping while using a screen; and using touchscreen devices were found to be associated with an increased risk of having a high problematic screen exposure score.

**Conclusion:**

Developing national scales to monitor problematic screen use in children would be more effective than monitoring screen time alone. All of the screen use characteristics not recommended in children would be evaluated using problematic screen exposure scales. The “Seven-in-Seven Screen Exposure Questionnaire” may serve as an example for further studies.

## Introduction

Screen media exposure has been increasing in the preschool years due to emerging technologies, growing marketing of digital media devices, increasing familial and societal use of screen media, and easy access to or ownership of screen media devices by younger children [1]. Watching television, digital video discs and videos, playing video games, and using apps are common types of screen exposure in children. Risky aspects of screen use such as excessive screen time, non-educational screen content, unsupervised screen use, rule-less screen use, and early onset of screen use have many potential negative effects on children’s health [1, 2]. In Turkey, 35.5% of children aged 6–9 years were reported to be engaged in screen time for ≥2 h per day and children aged 2–6 years were reported to spend an average of 86 min per day looking at screens [3, 4]. However, a past cross-sectional study among Turkish preschool children (3–6 years) reported an average of 2.2 h per day for viewing TV;  and half of them were in front of a screen without a parent or caregiver [5].

The determinants and relationships between developmental, behavioural, and health risks associated with screen media in pre-schoolers have become important issues in recent times [1, 6]. The reasons for focusing on this age group are as follows: (i) preschool children may form habits easily, and early excessive screen exposure increases the likelihood of excessive screen use in later life; (ii) health routines are established more easily in younger children than older ones; and (iii) screen use tends to increase over time, to experience more entertainment [6–11].

To promote health and development in preschool children, evidence-based guidelines have been developed for managing screen media usage. The main recommendations of these guidelines to parents/caregivers are minimising screen time, mitigating the risks associated with screen exposure, being mindful about the use of a screen, and demonstrating healthy screen use [11–13].

The American Academy of Pediatrics (AAP) reviewed the health and developmental concerns related to media use (obesity, shorter sleep duration, behavioural problems, and cognitive, language, and social-emotional delays) for children under 5 years old, and recommended management of young children’s media use in terms of time and content limits, and parent-child sharing. AAP recommendations set limits on media use in preschool-age children, such as avoiding digital media use in children younger than 18 to 24 months, choosing high-quality programming (educational, age-appropriate, and interactive), avoiding solo media use in 18- to 24-month-old children if digital media is introduced, limiting screen use to 1 h per day of high-quality programming, parent-child co-viewing for 2- to 5-year-old children, keeping mealtimes screen-free, and not using screen media for 1 h before bedtime [14].

In this digital age, physicians and other healthcare providers should consider asking screen exposure-related questions to parents/caregivers with young children in order to improve child health. While counselling parents/caregivers of preschool children on the recommended use of screen media, practical questionnaires may be useful. Starting from this point of view, we aimed to evaluate problematic screen media exposure in Turkish preschool children by using a unique tool called the “Seven-in-Seven Screen Exposure Questionnaire”, and to investigate factors associated with problematic screen media exposure. The “Seven-Seven Screen Exposure Questionnaire” can be used as a tool to identify young children and their families with inappropriate screen use and facilitate monitoring the results of preventive intervention efforts for them.

## Materials and methods

### Study design and study sample

This is a cross-sectional study that was carried out between July and December 2019 in three provinces in Turkey: Afyon, Ankara, and Mersin. Healthy, 2–6-year-old children who were admitted to the general paediatric clinics at three tertiary care hospitals in the three provinces for well-child examinations and their literate mothers were enrolled in this study. These three provinces are located in three different geographical regions of Turkey [15] and the hospitals where the study was conducted are health institutions that people of all socio-cultural and economic levels apply. Preschool children had a home-based care or a kindergarten care which remains open year-round. Mothers and/or children with chronic physical or psychiatric illness were excluded.

Sample size was 271 for each center (total: 1355), calculated with 50% for hypothesized frequency of outcome factor with 90% confidence interval and 1 for design effect with OpenEpi [16].

### Data collection

The mothers in the waiting room were invited to the study with the following statement: “We invite you to a survey investigating the screen media usage characteristics of your child. If you fill out a questionnaire form voluntarily, we will evaluate it and inform you about the outcomes.” The questionnaire and consent form were filled in by the mothers while waiting in line at the outpatient department. Subsequently, the filled forms were collected and the mothers were given counselling on problematic screen use during the well child examination.

### The participant survey form

A survey form was designed, including general descriptive questions and screen exposure characteristics according to published references [11, 12, 14]. The participant survey form was administered to 10 parents in each of five centres. Incomprehensible questions were reviewed with the study team. The modified questionnaire was applied to 20 parents in each center and it was seen that the questions were interpreted in the same way.

In the first part of the survey, family characteristics (parental ages and education levels, maternal occupation, family type and size, income level, settlement, province), child’s characteristics (age, gender, daycare, siblings) and screen use characteristics of the child (postponing eating, toileting or sleeping during screen use; having their own electronic screen device; using touch screen devices; video gaming), were collected as descriptive data. Parental education levels were categorized into ≤12 years and above compulsory. Family income level and settlement type were categorized according to the Turkish Statistical Institute classifications [15]. Child’s anthropometric measurements were obtained by the authors using regularly calibrated equipment and recorded on the survey form. Weight-for-age (WFA), height-for-age (HFA), and weight-for-height (WFH) z-scores were calculated using the World Health Organization child growth standards [17].

In the second part of the survey, a questionnaire we designed called the “Seven-in-Seven Screen Exposure Questionnaire” was conducted to evaluate problematic screen exposure. Items were designed using the AAP recommendations for children’s media use [14]. Daily screen time was asked as daily average usage of screens (TV and other devices: smartphone, computer, tablet, touch screen, and game console), including home care and nursery care, separately, for both weekdays and weekends. The average daily screen time was calculated as [(TV weekday+“Other Screens” weekday) × 5 + (TV weekend+“Other Screens” weekend) × 2]/7 and scored as the overall average screen time; 0: < 1 h; 1: 1–2 h; 2: > 2 h. Viewing with parent(s) was asked as the frequency of co-viewing and scored as shared screen use; 0: always; 1: sometimes; 2: rarely ever. Setting screen limits was asked as: “Do you set screen limits? If you set them, does your child obey these limits?”, and scored according to the presence of limits and the child’s compliance with limits; 0: parent setting limits and child obeying limits; 1: parent not setting limits; 2: parent setting limits but child not obeying limits. Screen exposure during meals and in the hour before bedtime was asked and scored whether this occurs or not; 0: no; 1: yes. Age of onset of screen exposure was asked and scored as the age of first screen exposure; 0: ≥ 24 months; 1: 18–23 months; 2: 12–17 months; 3: < 12 months. Screen content was asked in an open-ended format; if the response included only age-appropriate educational and prosocial content, the viewing of low-quality content was scored as 0; if the response included fast-paced programmes, apps with distracting and/or violent content, or older child/adult-oriented content, viewing low-quality content was scored according to the volume of inappropriate content; 1: 1 kind of inappropriate content; 2: ≥ 2 different kinds of inappropriate contents. All screen exposure characteristics were asked regarding the last month. The designed questionnaire and scoring are shown in Tables 1 and 2.

**Table 1:** The questions of the Seven-in-Seven Screen Exposure Questionnaire

1. How much time does your child spend per a typical weekday looking at TV? per a typical weekday looking at other screen devices? per a typical weekend day looking at TV? per a typical weekend day looking at other screen device?	
2. What age did your child start using screen device?	
3. Does your child use screen device during meals? ☐ Yes, he/she does ☐ No, he/she does not	
4. Does your child use screen device at least for 1 h before bedtime? ☐ Yes, he/she does ☐ No, he/she does not	
5. What are the types of programs and the kinds of screen content your child is exposed to?	
6. Do you share screen media experiences with your child? ☐ Always ☐ Sometimes ☐ Rarely ever	
7. Do you set screen limits? If you set them, does your child obey these limits? ☐ I set screen limits and my child obeys these limits. ☐ I do not set screen limits. ☐ I set screen limits but my child does not obey these limits.

**Table 2:** Scoring of Seven-in-Seven Screen Exposure Questionnaire

Characteristics	Scoring			
	0	1	2	3
**Screen exposure rules**				
Daily screen time	< 1 h	1-2 h	> 2	
Viewing with parent(s)	Always	Sometimes	Rarely ever	
Setting screen limits	Parent setting limits and child obeying limits	Parent not setting limits	Parent setting limits but child not obeying limits	
**Screen exposure during daily routines**				
During meals	No	Yes		
At least for 1 h before bedtime	No	Yes		
**Screen exposure conditions**				
Age of onset of screen exposure	≥24 mo	18–23 mo	12–17 mo	< 12 mo
Viewing low quality content	No	1 kind of inappropriate content	≥2 kinds of inappropriate contents	

### Study ethics

Written informed consent was obtained from all participants before enrolment in the study. Study procedures were performed in accordance with the Declaration of Helsinki, and the Ethics Committee of Hacettepe University approved the study (HU; 2019/12–06).

### Statistical analysis

The data were analysed using the SPSS 21 statistics program.

The Kaiser-Meyer-Olkin (KMO) test was used to carry out factor analysis of the data to evaluate its suitability, and Bartlett’s test was carried out to test the correlation between questionnaire items. According to the factor structure obtained from principal component analysis, the KMO coefficient was 0.61 (0.62 for children aged 24–48 months and 0.59 for children aged 49–72 months), the Bartlett’s test value was (× 2) 535.028, and the *p*-value was < 0.001. The analysis found a three-factor structure, thus the questionnaire included three categories: “screen exposure rules”, “screen exposure during daily routines”, and “screen exposure conditions”. The questionnaire included seven questions, three in the category “screen exposure rules”, two in “screen exposure during daily routines”, and two in “screen exposure conditions”. The reliability of the questionnaire with Cronbach’s alpha was 0.49. The total problematic screen exposure (PSE) score (range 0–13) was generated by summing scores from the three categories. Total scores are classified into two categories: low (< 7) and high (≥ 7). The cut-off point was defined as “7”, which is the 85th percentile (corresponding to a z-score of 1) value of PSE scores. Higher scores indicate more problematic screen exposure.

The Kolmogorov-Smirnov test, the coefficient of variation, skewness, kurtosis and histograms were used to test data for normality. PSE scores were right-skewed and skewed data are reported as median and interquartile range (IQR). Number and percentage values were given for categorical variables. A chi-square test was used to check the differences in frequencies between the groups. Logistic regression was performed to search for independent parameters associated with PSE. Ankara and Mersin are metropolitan cities with a population over 1.000.000, while Afyon is not [15]. For this reason, Afyon was taken as a reference. Multiple logistic regression used to predict high PSE as a dependent factor and included family characteristics (parental ages of < 30 vs. ≥ 30 years, parental education levels of ≤12 vs. > 12 years, maternal occupation of working vs. not working, income level of high vs. middle and low, family type of nuclear vs. single parent/extended, family size of < 5 vs. ≥ 5 members, urban vs. rural settlement, and residence in Afyon vs. Ankara and Mersin), the child’s characteristics [age of 24–48 months vs. 49–72 months, female vs. male, maternal daycare vs. other, no siblings vs. having 1 or ≥ 2 sibling(s)] and screen use characteristics of the child (not postponing vs. postponing eating, toileting or sleeping during screen use; not owning vs. owning 1 or ≥ 2 electronic screen device(s); not using vs. using touch screen devices; not playing video games vs. < 1 h and ≥ 1 h playing video games daily) as independent factors. Odds ratios (ORs) were calculated at a confidence interval (CI) of 95%. The threshold of statistical significance was set at *p* < 0.05.

## Results

### General characteristics

In total, 1245 mother-child pairs participated in this study. The median age of the children was 3.9 years (IQR: 2.9–4.7, mean ± SD: 3.8 ± 1.0) years and 51% were males. Overall, 965 children (77.5%) had a PSE score of < 7 (low) and 280 children (22.5%) had a PSE score of ≥7 (high). The median PSE score was 4 (IQR: 3–6), and median scores were 2 (1–4), 0 (0–1), and 2 (0–3) in the “screen exposure rules”, “screen exposure during daily routines”, and “screen exposure conditions” categories of the PSE questionnaire, respectively.

### Problematic screen exposure characteristics

The frequency of high PSE scores significantly varied with maternal age, parental educational levels, maternal occupation, family type and size, and settlement. Maternal age of ≥30 years and parental educational level of > 12 years were associated with a decreased risk of having a high PSE score (*p* < 0.05). Non-working mother, single parent or extended family, family size of ≥5 members, and rural settlement were associated with an increased risk of having a high PSE score (*p* < 0.05). Children using a kindergarten daycare had a decreased risk for having a high PSE score compared to children using home-based daycare (*p* < 0.01). Children who have ≥2 siblings had an increased risk of having a high PSE score than children who have no siblings (*p* < 0.001). Table 3 shows the associations between family-child characteristics and having a high PSE score.

**Table 3:** Associations between family, child, and screen use characteristics and having high PSE score

	Overall (***n*** = 1245) N (%*)	High PSE score (≥7) %**	***p***-value	OR (95%CI)	AOR (95%CI)^**#**^
**Family characteristics**					
Maternal age					
< 30 years	313 (25.1)	28.8	**0.002**	1.00	1.00
≥ 30 years	932 (74.9)	20.4		0.63 (0.47–0.85)	0.63 (0.43–0.94)
Paternal age					
< 30 years	126 (10.1)	20.6	0.599	1.00	1.00
≥ 30 years	1119 (89.9)	22.7		1.13 (0.72–1.79)	2.12 (1.18–3.82)
Maternal education					
≤ 12 years	297 (23.9)	36.4	**< 0.001**	1.00	1.00
> 12 years	948 (76.1)	18.1		0.39 (0.29–0.52)	0.59 (0.38–0.92)
Paternal education					
≤ 12 years	226 (18.2)	33.6	**< 0.001**	1.00	1.00
> 12 years	1019 (81.8)	20.0		0.49 (0.36–0.68)	0.91 (0.60–1.40)
Maternal occupation					
Working	603 (48.4)	15.8	**< 0.001**	1.00	1.00
Not working	642 (51.6)	28.8		2.17 (1.64–2.86)	1.25 (0.77–2.04)
Income level					
High	696(55.9)	21.0	0.305	1.00	1.00
Middle	321 (25.8)	25.2		1.27 (0.93–1.74)	1.14 (0.80–1.62)
Low	228 (18.3)	23.2		1.14 (0.80–1.63)	0.97 (0.65–1.47)
Family type					
Nuclear	1076 (86.4)	21.1	**0.003**	1.00	1.00
Single parent or extended	169 (13.6)	31.4		1.71 (1.20–2.44)	1.61 (0.94–2.74)
Number of family members					
< 5	940 (75.5)	19.3	**< 0.001**	1.00	1.00
≥ 5	305 (24.5)	32.5		2.02 (1.51–2.69)	0.90 (0.46–1.77)
Settlement					
Urban	930 (74.7)	20.3	**0.002**	1.00	1.00
Rural	315 (25.3)	28.9		1.59 (1.19–2.13)	1.27 (0.89–1.80)
Province					
Afyon	205 (16.5)	17.6	0.152	1.00	1.00
Ankara	861 (69.1)	23.8		1.47 (0.99–2.17)	2.54 (1.60–4.04)
Mersin	179 (14.4)	21.8		1.31 (0.79–2.17)	1.72 (0.98–3.05)
**Child characteristics**					
Age group					
24–48 months	647(52.0)	24.6	0.067	1.00	1.00
49–72 months	598(48.0)	20.2		0.78 (0.60–1.02)	0.71 (0.51–0.99)
Gender					
Female	610 (49.0)	20.8	0.167	1.00	1.00
Male	635 (51.0)	24.1		1.21 (0.92–1.58)	1.10 (0.81–1.48)
Daycare of the child					
Mother	561 (45.1)	30.1^a^	**< 0.001**	1.00	1.00
Granparent	200 (16.1)	21.5^b^		0.64 (0.43–1.19)	0.96 (0.53–1.75)
Childminder	58 (4.6)	20.7^b^		0.61 (0.31–1.17)	1.03 (0.45–2.38)
Kindergarten	426 (34.2)	13.1^c^		0.35 (0.25–0.49)	0.60 (0.36–0.99)
Number of sister/brother					
0	442 (35.5)	17.4^a^	**< 0.001**	1.00	1.00
1	580 (46.6)	21.7^a^		1.32 (0.96–1.80)	1.10 (0.77–1.57)
≥ 2	223 (17.9)	34.5^b^		2.50 (1.73–3.62)	2.04 (0.98–4.21)
**Screen use characteristics**					
Postponing eating or toileting or sleeping while screen using					
Never	846 (67.9)	16.1^c^	** < 0.001**	1.00	1.00
Sometimes	280 (22.5)	30.7^b^		2.31 (1.69–3.16)	4.43 (2.86–6.85)
Frequently	119 (9.6)	48.7^a^		4.96 (3.31–7.43)	2.73 (1.94–3.85)
Having own electronic screen device					
None	979 (78.6)	23.6	0.118	1.00	1.00
1 device	238 (19.1)	19.3		0.78 (0.55–1.11)	0.87 (0.59–1.28)
≥ 2 devices	28 (2.3)	10.7		0.39 (0.12–1.30)	0.42 (0.12–1.48)
Using touchscreen devices					
No	403(32.4)	16.4	**0.001**	1.00	1.00
Yes	842(67.6)	25.4		1.74 (1.28–2.36)	1.68 (1.19–2.38)
Daily duration of video gaming					
None	915 (73.5)	22.2^a^	**0.006**	1.00	1.00
< 1 h	239 (19.2)	18.8^a^		0.81 (0.57–1.17)	0.86 (0.58–1.28)
≥ 1 h	91 (7.3)	35.2^b^		1.90 (1.20–3.00)	1.56 (0.93–2.64)
Antropometric data					
WFH z-score					
< −2.0	62 (5.0)	21.0	0.837	1.00	
-2.0, 2.0	821 (65.9)	22.5		1.10 (0.58–2.06)	
> 2.0	80 (6.4)	26.3		1.34 (0.61–2.95)	
Missing	282 (22.7)	21.6		1.04 (0.53–2.04)	
HFA z-score					
< −2.0	52 (4.2)	26.9	0.879	1.00	
-2.0, 2.0	806 (64.7)	22.5		0.79 (0.42–1.48)	
> 2.0	111 (8.9)	22.5		0.79 (0.37–1.68)	
Missing	276 (22.2)	21.7		0.75 (0.38–1.48)	
WFA z-score					
< −2.0	16 (1.3)	31.3	0.229	1.00	
-2.0, 2.0	904 (72.6)	22.0		0.62 (0.21–1.81)	
> 2.0	44 (3.5)	34.1		1.14 (0.33–3.88)	
Missing	281(22.6)	21.8		0.61 (0.20–1.82)	

*column percentage; **row percentage

*WFH* Weight for height, *HFA* height for age, *WFA* weight for age

^abc^Values with different superscripts differ significantly in subgroup analysis (*p* < 0.05)

# adjusted for included family characteristics, child characteristics, screen use characteristics of children

Children who postpone essential needs while using a screen, use touchscreen devices, and play video games for ≥1 h per day had an increased risk of having a high PSE score (*p* < 0.01, Table 3). The frequency of high PSE scores did not significantly vary with the anthropometric z-score groups (*p* > 0.05, Table 3).

Multiple logistic regression revealed that maternal age of < 30 years, paternal age of ≥30 years, maternal educational level < 12 years, residence in Ankara, and postponing essential needs while using a screen and using touchscreen devices were associated with an increased risk of having a high PSE score. However, kindergarten daycare was associated with a decreased risk of having a high PSE score. Adjusted ORs (95% CI) are shown in Table 3.

## Discussion

In this study, a tool called the “Seven-in-Seven Screen Exposure Questionnaire” was designed by referencing AAP recommendations and used to evaluate PSE in Turkish preschool children. By using this novel scale, parental ages, maternal education level, child’s age, daycare type, and use of touchscreens were demonstrated to be associated with an increased risk of having a high PSE score. Originally, we determined that postponing essential needs while using a screen is associated with an increased risk of having a high PSE score in pre-schoolers.

Screen media devices have become an inseparable part of daily life, and their use can begin in early childhood. Excessive/inappropriate use of screen media devices may become problematic in children, and this potentially problematic usage may culminate in addiction to screen media devices. Sleeping less, losing interest in other activities, not being able to control use of a digital device, feeling the need to spend more time using a digital device, and the idea of using digital devices as being the most important thing in life are some of the items on The Digital Addiction Scale for Children [18, 19]. Therefore, the finding obtained in the present study that the risk of having a high PSE score increases when a child is postponing essential needs while using a screen should be emphasised in the guidelines. We suggest that postponing essential needs while using a screen indicates PSE in preschool age children, and may be a warning sign of future screen media addiction. History of the child’s screen usage should include asking about screen addiction vulnerability.

Excess screen time in preschool children has a prevalence of 10 to 93% in high-income countries and 21 to 98% in middle-income countries, and it is associated with various health risks [6]. Therefore, the burden of problematic screen exposure seems significant worldwide. When associated factors and correlates of screen time were reviewed, parental and familial demography, birth order and daycare of the child, and digital media micro and macro environment were found to be associated with the child’s screen time [6]. Also, similar associations were found in this study in terms of PSE by scoring daily screen time according to its volume. Association between lower parental education and excessive screen time is known [5, 20, 21]. Yalcin et al. reported a negative correlation between TV viewing time and paternal education [5]. In a sample of Greek preschool children, it was found that maternal educational status and region of residence were significantly associated with children’s screen time. Maternal education level of > 12 years and small-town residence were found to be associated with decreased risk of having excessive screen time [22]. We also found a similar result in terms of maternal educational status. Although we found no association between settlement type and having a high PSE score, we demonstrated that living in Ankara was associated with having a high PSE score. Ankara, the capital of Turkey, is a larger province than Afyon and Mersin, so we can suggest that small province residence may be associated with a decreased risk of having PSE compared to large province residence. An early childhood longitudinal birth cohort study reported that screen time was related to daycare of the child; children in home-based care had higher screen time than children cared for in child-care centres [23]. Similarly, we found that kindergarten daycare was associated with decreased risk of having PSE compared to home-based daycare. Parental ages were also found to be associated with the child’s PSE in this study. In addition to parental ages, parental and familial demographic characteristics were also investigated as associated factors of screen time in previous studies, with inconsistent findings [6]. The associations found in this study contribute to the existing literature by evaluating problematic screen use, which includes daily screen time.

It is known that parents play a crucial role in providing their children with appropriate screen use. In order to fulfill this parental duty, Wu et al. suggested that parents should practice a supervisory (combination of restrictive and instructive) and co-using approach [24]. Besides, family-based interventions to reduce screen time in preschool children have shown significant effectiveness in several countries [6]. From this point of view, including screen exposure rules nominately parent-child co-viewing and setting screen limits in the “Seven-in-Seven Screen Exposure Questionnaire” broadens perspective in evaluating problematic screen use. In previous studies, often or always limiting child’s TV use and using screen time rules were found to be associated with lower screen time [25, 26]. Therefore, including the setting of screen limits in our unique questionnaire and scoring it by considering the child’s compliance can provide a useful point of view for evaluation of problematic screen use.

Screen exposure during dinner or lunch and within 1 h of bedtime was found to be positively associated with increased screen time in children under five [26–28], and increasing screen time indicates increasing PSE score according to our unique questionnaire. Screen media use during family meals may reduce family interactions, and screen media use in the hour before bedtime may reduce shared parent-child experiences such as reading books. If screen use causes parent-child conflict and distraction in parent-child engagement, this is problematic screen use. Also, not keeping mealtimes screen-free may impair satiety, and not keeping bedtimes screen-free may impair sleep quality and quantity [1, 14]. Therefore, including screen exposure during daily routines in the “Seven-in-Seven Screen Exposure Questionnaire” is useful for evaluating problematic screen use.

The age of first screen exposure was around 4 years of age around five decades ago, but today it is 4 months of age [29]. However, the AAP does not recommend screen media use, other than video chatting, for children < 18 months of age [14]. Developing addictive behaviour, impairing vocabulary acquisition, and deterioration of language, cognitive, and executive skills are potential negative effects of early screen exposure, due to the developing brain being at its most vulnerable stage [30]. Early age of initiation of electronic screen exposure causes high screen time in the following years and decreased caregiver-child interaction [4]. Therefore, including the age of onset of screen exposure in our unique questionnaire and scoring it by considering younger ages at initial screen exposure is justified for evaluation of problematic screen use.

A neglectful parenting style has been reported to be associated with higher child screen time [31]. Therefore, we might assume that viewing low-quality media content would be associated with problematic screen use, as not avoiding inappropriate media content while guidelines recommend it is considered to be neglect. Also, parental TV content rules were found to be associated with a lower likelihood of falling outside the AAP guidelines [32]. Therefore, including the viewing of low-quality content in our unique questionnaire and scoring it by considering the volume of inappropriate content is a valid component of the evaluation of problematic screen use.

Age and gender were investigated as possible associated factors and correlates of screen time in several studies. Hinkley et al reported no relationship between gender and screen time in preschool children [33]; we also demonstrated no association between gender and PSE. On the other hand, previous studies reported a positive relationship between age and screen time in preschool children [22, 33–35], while this study found that an age of 49–72 months old was associated with a decreased risk of having a high PSE score compared to children aged 24–48 months old. First of all, to discuss these previous findings, we must remember that the PSE score was the sum of the scores of seven items, so the daily screen time score alone does not reflect PSE. The age of onset of mobile device (smartphones and tablet computers) use is getting younger in children [36], so a younger child may have a higher PSE score due to a higher screen exposure conditions score. Another suggestion is that parents may bend or remove screen use limits and use media to calm their younger children, which results in higher levels of screen exposure rules and PSE scores in the younger age group. We believe that there is a need to develop problematic screen exposure scales in national settings, with clarification of the specific rules for different stages of childhood: infancy, toddlerhood, preschool age, school age, and adolescence.

In recent years, mobile screen media exposure and independent activity on mobile devices have been increasing among young children. In the United States, among 0- to 8-year-olds, average daily time spent using a mobile device was 5, 15, 48 and 55 min in 2011, 2013, 2017 and 2020, respectively [37]. Kabali et al. reported that at age 4, three-fourths of U.S. children had their own mobile device, almost all children (96.6%) used mobile devices, most started using before age 1, and mobile screen time and independent use of mobile devices increased with age in young children [38]. Because of the increasing ownership, availability, and use of touchscreen media devices, total daily screen time has increased and parental control and media use rules have decreased in young children [7]. We also found that use of touchscreen devices is associated with an increased risk of PSE in preschool age children. Therefore, it is important to give parents guidance about allowing their young children to use touchscreen media devices under adult supervision.

A recent systematic review and meta-analysis concluded that increasing screen time could be a risk factor for being overweight/obese in children and adolescents [39]. An increase in screen time, indicating an increase in PSE score, was one of the factors of our unique questionnaire, but we found no association between having a high PSE score and anthropometric z-scores. This cross-sectional study is descriptive, not causal or relational; hence, further longitudinal studies should investigate the association between being overweight/obesity and problematic screen use, as well as excessive screen time.

Previous studies have mostly focused on factors that influence screen time in preschool children [8, 40–42]. However, we explored factors influencing problematic screen exposure, considering not only daily screen time, but also parent-child co-viewing, screen limit settings, screen usage at bedtime and mealtimes, age of media use onset, and screen media content. Moreover, we investigated this using a suitable scale and scoring system, which is the main strength and originality of our study. Through including children from three different provinces in three different geographic regions of our country, our results represent the national epidemiology of PSE in preschool children.

There are several limitations of this study. First, our questionnaire is incomplete, as we did not include questions on turning off screen devices when not in use, which is one of the current AAP recommendations on screen use. Second, even though the AAP recommends children younger than 18 months have no screen use except video-chatting, we scored being exposed to screens at < 18 months of age, without considering the type of screen use, as there is no reported benefit of engaging in screen use in children younger than 18 months of age, while there are reported potential adverse effects [30]. Third, even though the AAP recommends that 18–24-month-old children may use a screen with high-quality programming/apps with active adult interaction, we also scored being exposed to screens at 18–23 months of age without considering content and type of screen use, because the evidence for benefits of screen use is still limited in children younger than 2 years [14]. However, we accept that the lack of certain benefits does not indicate problematic screen use. Further studies on problematic screen use in children aged 2–5 years should review scoring of the age of first screen exposure by taking into account that parents can erroneously report their child’s previous screen use, as recall errors surrounding the content and type of screen exposure in infancy and toddlerhood may result in underestimates of problematic screen use. Fourth, if participant mothers are aware of what is considered appropriate screen use, a self-reporting bias could occur. Therefore, further studies are needed to develop a diagnostic tool for reliably evaluating a child’s problematic screen exposure and its short- and long-term consequences.

## Conclusion

This study investigating the factors associated with problematic screen exposure revealed findings consistent with previous studies investigating the factors associated with excessive screen time. Excessive screen time is not the only factor involved in PSE. The other not recommended screen use characteristics such as independent and unrestricted use, use during meals and before bedtime, use under 2 years of age, and not using high-quality programming should be considered, in addition to excessive screen time. Developing national scales to monitor problematic screen use in children would be more effective than monitoring screen time alone. All of the screen use characteristics not recommended in children would be evaluated using PSE scales. Problematic screen exposure scales might be useful for making guidelines for limiting problematic screen use, formulating culturally-appropriate intervention strategies, and monitor the results of intervention studies. Also, such scales may make it easier for paediatricians to monitor this issue, while exploring the history of screen usage from the parents. The “Seven-in-Seven Screen Exposure Questionnaire” may serve as an example for further studies.

## Data Availability

Data can be requested from corresponding author.
